# Reconfigurable carrier type and photodetection of MoTe_2_ of various thicknesses by deep ultraviolet light illumination†

**DOI:** 10.1039/d1na00881a

**Published:** 2022-05-10

**Authors:** Byung Min Ko, Muhammad Farooq Khan, Ghulam Dastgeer, Gyu Nam Han, Muhammad Asghar Khan, Jonghwa Eom

**Affiliations:** Department of Physics & Astronomy and Graphene Research Institute, Sejong University Seoul 05006 Korea eom@sejong.ac.kr; Department of Electrical Engineering, Sejong University 209 Neungdong-ro, Gwangjin-gu Seoul 05006 Korea

## Abstract

Tuning of the Fermi level in transition metal dichalcogenides (TMDCs) leads to devices with excellent electrical and optical properties. In this study, we controlled the Fermi level of MoTe_2_ by deep ultraviolet (DUV) light illumination in different gaseous environments. Specifically, we investigated the reconfigurable carrier type of an intrinsic p-MoTe_2_ flake that gradually transformed into n-MoTe_2_ after illumination with DUV light for 30, 60, 90, 120, 160, 250, 500, 900, and 1200 s in a nitrogen (N_2_) gas environment. Subsequently, we illuminated this n-MoTe_2_ sample with DUV light in oxygen (O_2_) gas and reversed its carrier polarity toward p-MoTe_2_. However, using this doping scheme to reveal the effect of DUV light on various layers (3–30 nm) of MoTe_2_ is challenging. The DUV + N_2_ treatment significantly altered the polarity of MoTe_2_ of different thicknesses from p-type to n-type under the DUV + N_2_ treatment, but the DUV + O_2_ treatment did not completely alter the polarity of thicker n-MoTe_2_ flakes to p-type. In addition, we investigated the photoresponse of MoTe_2_ after DUV light treatment in N_2_ and O_2_ gas environments. From the time-resolved photoresponsivity at different polarity states of MoTe_2_, we have shown that the response time of the DUV + O_2_ treated p-MoTe_2_ is faster than that of the pristine and doped n-MoTe_2_ films. These carrier polarity modulations and photoresponse paves the way for wider applications of MoTe_2_ in optoelectronic devices.

## Abstract 2

We have modulated the Fermi level of MoTe_2_ by deep ultraviolet light (a wavelength of 220 nm) illumination in different gaseous environments.

## Graphical abstract

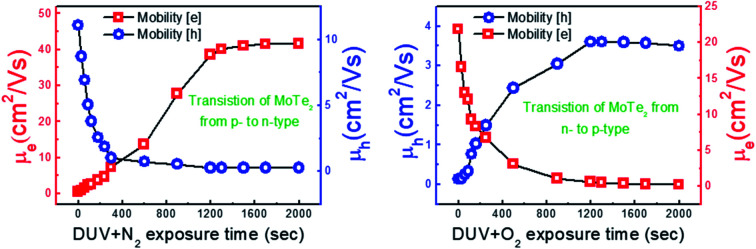

## Introduction

Recently, two-dimensional (2D) nanomaterials have gained significant attention because of their unique optical and electrical properties.^1–3^ Graphene has been the focus of modern research in the past few years because of its high intrinsic electron mobility of 200 000 cm^2^ V s^−1^ and high thermal conductivity of approximately 4400 to 5780 W m K^−1^ at room temperature.^4,5^ In addition, the transmittance of light was found to be 97.7% at 550 nm owing to the small thickness of a single layer.^6^ However, the absence of the bandgap in graphene initiates the search for other 2D bandgap materials, which is necessary for switching device applications. Transition metal dichalcogenides (TMDCs) have a significant bandgap range of approximately 1.0 to 2.5 eV, which facilitates fabrication of atomically thin p–n diodes such as MoTe_2_/MoS_2_, WSe_2_/MoS_2_ and black phosphorus/IGZO.^7–9^ The Fermi level of TMDCs is near the center between conduction and valence bands,^10^ but most TMDCs used in experiments are naturally n-type or p-type doped due to intrinsic structural defects and interface impurities.^11,12^

Controlled modulation of the Fermi level in the bandgap of TMDCs has been exploited in various reports for device applications.^13,14^ While most of the well-known TMDCs such as WS_2_, MoS_2_, MoSe_2_, ReSe_2_, and ReS_2_ are observed to be n-type semiconductors, MoTe_2_ is p-type when thin, and shows ambipolar and n-type properties in thicker forms.^15–19^ In addition, MoTe_2_ has shown great potential in applications, such as chemical sensors, memristors, and photodetectors.^20–22^ The thicker hexagonal structure of MoTe_2_ has a bandgap of 0.88 eV, whereas its mono-layer exhibits a direct bandgap of almost 1.1 eV.^23,24^ The narrow bandgap also enables a huge tunneling current and a high on-off ratio of MoTe_2_ FETs. The pinning of the Fermi level at the MoTe_2_-metal interface is not as strong as that of sulfur-terminated TMDCs.^25^ Thus, the weak pinning and small bandgap of MoTe_2_ make the Fermi level modulation feasible and carrier polarity controllable by various techniques. The doping treatment of MoTe_2_ has been accomplished by electrostatic gating, chemical doping, and work function engineering with different metal electrodes; however, the doping effect was weak and too volatile for electronic devices.^26–28^ Designing a constructive approach that provides non-volatility and reversibility of charge carrier polarity in 2D TMDC materials is indispensable. Therefore, MoTe_2_ with layers of different thicknesses is a promising candidate for overcoming the abovementioned challenges. However, there is still ambiguity in the doping effect and carrier-type inversion of MoTe_2_ layers with different thicknesses when exposed to deep ultraviolet (DUV) light in various gaseous environments.

In this study, we fabricated a MoTe_2_ field-effect transistor (FET) on a SiO_2_ substrate using the mechanical exfoliation technique. We exposed MoTe_2_ thin-film FETs under deep ultraviolet (DUV) light for different illumination times in the presence of different gases (N_2_ or O_2_), which resulted in a non-volatile doping effect and inversion of charge carrier polarity in MoTe_2_ FETs. In addition, we investigated the photodetection of DUV light after a variety of doping treatments (pristine p-MoTe_2_, DUV + N_2_ treatment, and DUV + O_2_ treatment).

## Fabrication

**Fig. 1:**
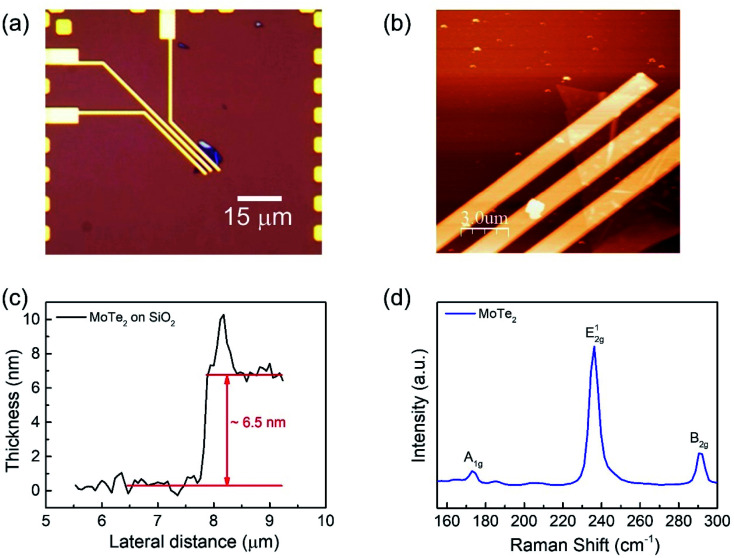
(a) Optical microscope image of the MoTe_2_ device. (b) Atomic force microscopy (AFM) image of the MoTe_2_ device. (c) The thickness profile of the MoTe_2_ thin film by AFM. (d) The Raman spectroscopy image of the MoTe_2_ film under ambient conditions.

We fabricated a MoTe_2_ FET on a SiO_2_ substrate (Fig. 1a) to elucidate the carrier polarity modification. Bulk MoTe_2_ was first put on tape and then exfoliated to make the MoTe_2_ thin films.^29^ Then, the tape was pressed on top of polydimethylsiloxane (PDMS). Subsequently, PDMS was stamped on the desired area on the Si/SiO_2_ substrates.^30^ A MoTe_2_ thin film was produced as confirmed using optical microscopy. Then, conventional e-beam lithography was employed to make Cr/Au (5 nm/60 nm) electrical contacts, which were annealed at 100 °C for 1.5 h to improve the ohmic properties.^30–32^ The optical microscope images of the final device are shown in Fig. 1a. Meanwhile, the thickness of the flakes was investigated using atomic force microscopy (AFM). Fig. 1b and c show the AFM images of the film and the thickness of the exfoliated MoTe_2_ film was found to be approximately 6.5 nm.^15,33^ Then, the crystal properties of MoTe_2_ were examined by Raman spectroscopy^15^ as shown in Fig. 1d.

Furthermore, the DUV doping treatments were performed in N_2_ and O_2_ gas environments at a pressure of 0.8 bar. The average intensity of the DUV light was 11 mW cm^−2^, which was measured at a wavelength of 220 nm.

## Results and discussion

### Transition from intrinsic p-type MoTe_2_ to n-type MoTe_2_

Raman spectroscopy was performed to examine the crystal bonding properties and estimate the thickness of the pristine MoTe_2_ flakes. Fig. 1d shows the Raman spectrum of MoTe_2_ obtained using a Renishaw microspectrometer with a laser wavelength of 514 nm and an optical intensity of 1 mW cm^−2^. Three vibrational modes were observed, namely, A^1^_g_, E^2g^_1_ and B^2g^_1_ at approximately 173.8 cm^−1^, 236.2 cm^−1^, and 291.1 cm^−1^, respectively. These results reveal five layers of MoTe_2_, which is almost consistent with previous reports.^34,35^

**Fig. 2:**
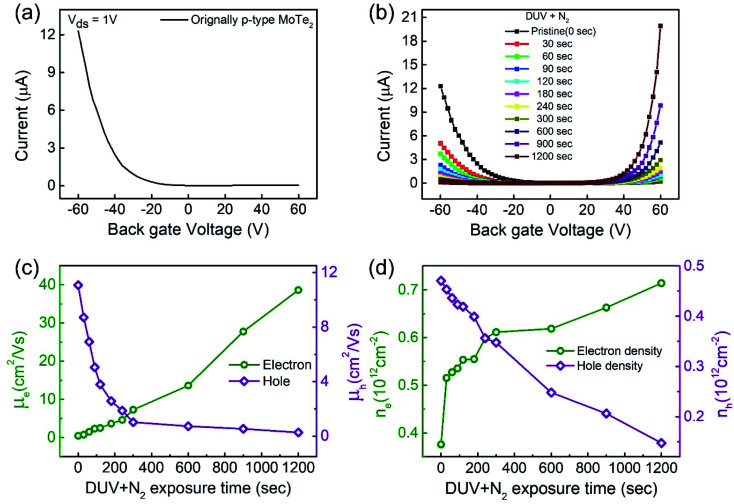
(a) Transfer curve (*I*_ds_–*V*_g_) of the intrinsic p-MoTe_2_ device. (b) The transfer curves at different DUV + N_2_ treatment times. (c) Electron and hole mobility at different DUV + N_2_ treatment times. (d) The charge carrier density of electrons and holes of the MoTe_2_ flake for various DUV + N_2_ treatment times.

Furthermore, we investigated the intrinsic nature of the charge carriers of the MoTe_2_ flakes by measuring the transfer curves (*I*_ds_*vs. V*_g_) at a fixed source–drain bias voltage *V*_ds_ = 1 V. After sweeping the gate voltage from −60 to 60 V, a threshold current (*I*_ds_) was observed in the negative gate voltage regime, confirming the p-type nature of the pristine MoTe_2_ flake, as shown in Fig. 2a. Subsequently, we treated the pristine p-MoTe_2_ with DUV light in a N_2_ environment (DUV + N_2_) for 1200 s to obtain n-MoTe_2_. The change in the charge carrier type was confirmed by using the *I*_ds_–*V*_g_ transfer curves, as shown in Fig. 2b. The DUV exposure time was varied from 0 to 1200 s, with a time interval of 30 s. The DUV + N_2_ treatment gave rise to n-type doping in MoTe_2_. The Fermi level of the intrinsic p-type MoTe_2_ was near the valence band, but after the DUV + N_2_ treatment, the Fermi level shifted near the conduction band. This Fermi level shift is responsible for the change in the nature of the MoTe_2_ flakes from p- to n-type. To verify the doping mechanism more precisely, the mobility of electrons/holes of intrinsic p-MoTe_2_ and n-MoTe_2_ (after DUV + N_2_ treatment) sheets was extracted from the transfer curves using the following equation:1
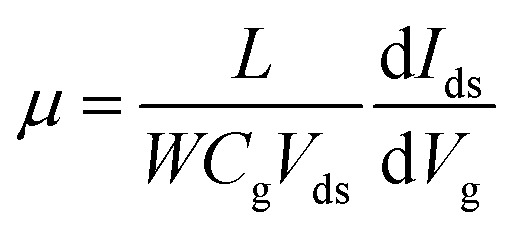
Here, *L* (*W*) is the length (width) of the active channel area of MoTe_2_ nano-sheets, *C*_g_ = 11.5 nF cm^−2^, is the gate capacitance,^36^ and *V*_ds_ (*I*_ds_) is the source–drain voltage (current). In this work, we used a MoTe_2_ device with *L* = 1 μm and *W* = 5.6 μm, whereas the source–drain voltage was fixed at 1 V. Notably, the hole mobility gradually decreased from 11 cm^2^ V^−1^ s^−1^ to almost zero, while the electron mobility began to increase from 0.1 to 40 cm^2^ V^−1^ s^−1^ as the MoTe_2_ flakes underwent DUV + N_2_ treatment, as shown in Fig. 2c. This treatment made the doping of intrinsic p-MoTe_2_ and changed the carrier type from p-type to n-type after undergoing ambipolar behavior. The sample finally transformed into n-MoTe_2_ as the exposure time reached 1200 s.

Moreover, the charge carrier density of holes in p-type MoTe_2_ and electrons in DUV + N_2_ treated MoTe_2_ sheets was calculated using the relation:^37,38^

2 *n* = *q*^−1^*C*_g_|*V*_th_ − *V*_g_|

where *q* = 1.6 × 10^−19^ C is the charge of the electron, *C*_g_ = 11.5 nF cm^−2^ is the gate capacitance, *V*_th_ is the threshold voltage observed in the transfer curve, and *V*_g_ is the applied gate voltage (±40 V). Therefore, we can determine the carrier density of MoTe_2_ according to the change in the threshold voltage. When the DUV + N_2_ exposure time was zero, the electron density was significantly small (3.7 × 10^11^ cm^−2^) compared to the hole density (7.4 × 10^11^ cm^−2^), and the dominant carrier type was reversed over time. Therefore, the N_2_ gas under DUV light removes oxygen on the surface of MoTe_2_, which alters its intrinsic p-type nature to n-type MoTe_2_, as shown in Fig. 2d.

### Transition from n-type MoTe_2_ to p-type MoTe_2_

**Fig. 3:**
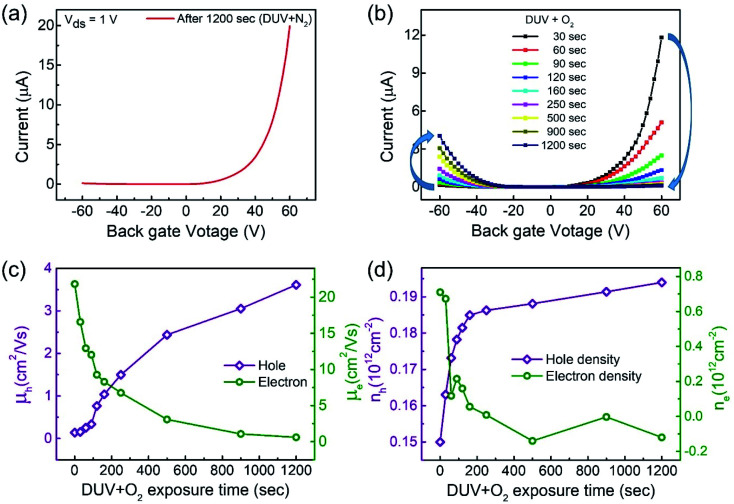
(a) Transfer curve of the MoTe_2_ device after DUV + N_2_ doping for 1200 s. (b) Transfer curves after DUV + O_2_ treatments for various times. (c) Electron and hole mobility for various DUV + O_2_ treatment times. (d) Charge carrier density of electrons and holes of the MoTe_2_ flake for various DUV + O_2_ treatment times.

Interestingly, the n-MoTe_2_ flake, which was treated with DUV + N_2_ for 1200 s, could be reconfigured to p-MoTe_2_ by DUV + O_2_ treatment. As mentioned previously, the source–drain voltage was fixed at *V*_ds_ = 1 V, and the back-gate voltage was applied to the substrate to check the *I*_ds_–*V*_g_ transfer curve from 60 V to −60 V. Fig. 3a shows the transfer curve of n-MoTe_2_ treated with DUV + N_2_ for 1200 s. Then, the transfer curves were measured as we increased the treatment time of DUV + O_2_, as shown in Fig. 3b. The mobility of the electrons (holes) in the n-MoTe_2_ sheets was estimated to be 0.25 cm^2^ V^−1^ s^−1^ (3.8 cm^2^ V^−1^ s^−1^) after the DUV + O_2_ treatment, as shown in Fig. 3c. As the exposure time of the DUV + O_2_ treatment increased from 0 to 1200 s, the mobility of electrons decreased, while the mobility of holes increased. Similarly, the carrier density of the holes (electrons) at a fixed gate voltage of ±40 V increased (decreased) with increasing exposure time, as shown in Fig. 3d. Initially, the carrier density increased abruptly but started to saturate with time after 200 s. The increase in the hole density indicates that the nature of n-type MoTe_2_ shifted back to that of p-type MoTe_2_ after the DUV + O_2_ treatment. This reverse doping could be ascribed to the oxygen donor molecules under DUV light, and the shift of the MoTe_2_ Fermi level near the valence band. In case of transition from pristine p-MoTe_2_ to n-MoTe_2_, the current and mobility values are saturated after 1200 s DUV + N_2_ treatment as shown in Fig. S2a and S2b (see the ESI†). On the other hand, when n-type doped MoTe_2_ (self-made) is converted to p-MoTe_2_ by DUV + O_2_ treatment, the current and mobility values reach the maximum values, but after 1200 s they slightly start to degrade due to weak oxidation of MoTe_2_ under DUV + O_2_ treatment (see Fig. S1c and S1d†).

**Fig. 4:**
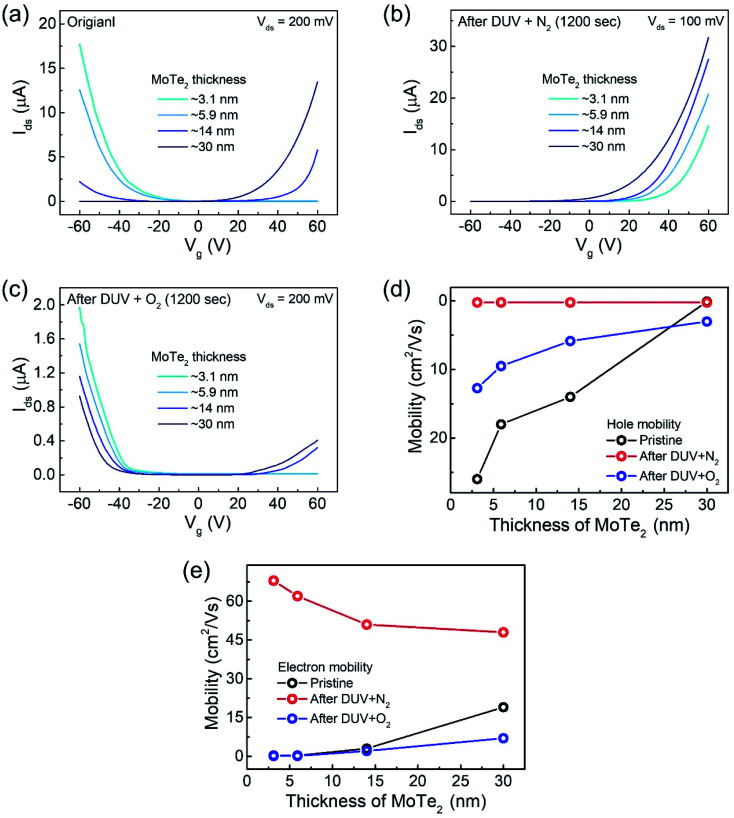
Transfer curves of (a) pristine p-MoTe_2_ flakes. (b) DUV + N_2_ treated MoTe_2_ flakes. (c) DUV + O_2_ treated MoTe_2_ flakes. (d) Hole mobility of all devices having different thicknesses of MoTe_2_. (e) Electron mobility of all devices with different thicknesses of MoTe_2_.

In addition, we investigated several intrinsic p-MoTe_2_ devices with different flake thickness values under DUV light in N_2_ and O_2_ environments. AFM images and thickness profiles of the devices are shown in Fig. S2.† As shown in Fig. 4a, we measured the *I*_ds_–*V*_g_ curves of intrinsic MoTe_2_ devices with different thicknesses (3.1, 5.9, 14, and 30 nm). However, we found that the thin flake devices showed only p-type behavior, whereas the thicker flakes showed ambipolar or n-type characteristics. After the DUV + N_2_ treatment, all devices were then completely configured into n-type, as shown in Fig. 4b. Subsequently, we exposed the n-MoTe_2_ devices to DUV + O_2_ treatment, which altered all the n-type devices to p-type. Interestingly, only the thin devices completely changed their polarities. The thicker devices did not completely change back to p-type but showed ambipolar behavior, as shown in Fig. 4c. The DUV + O_2_ treatment did not fully dope the thick flakes. Furthermore, the mobilities of electrons and holes for all devices (pristine, after DUV + N_2_, and DUV + O_2_ treatments) were estimated, as shown in Fig. 4d and e. While the 3.1 nm-thick MoTe_2_ flake was p-type in the pristine state, the 30 nm-thick MoTe_2_ flake was n-type in the pristine state. We found that the DUV treatments were more efficient for thinner MoTe_2_ flakes.

### Photodetector measurements

**Fig. 5:**
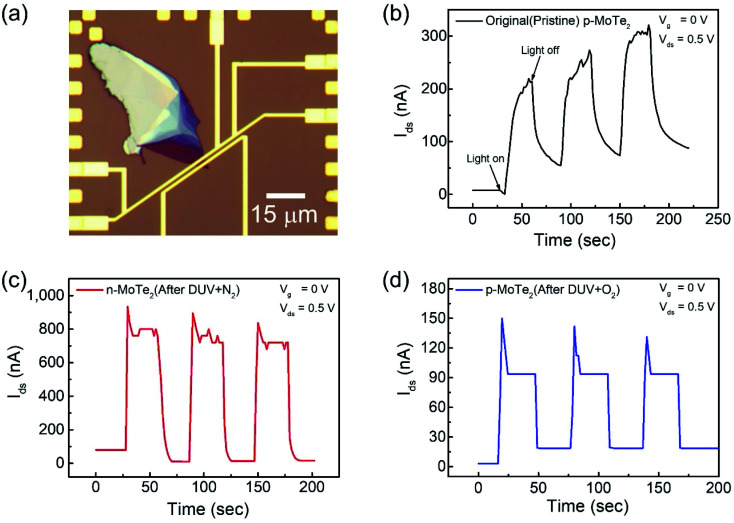
(a) Optical microscope image of the pristine p-MoTe_2_ device. Photoresponse of (b) pristine p-MoTe_2_, (c) after 1200 s DUV + N_2_ treatment and (d) after 1200 s DUV + O_2_ treatment.

The key factors in photodetection are current photo-generation and photoresponsivity, which play promising roles in optical devices. Therefore, the time-resolved photoresponse of the fabricated FETs was examined under a DUV lamp (*λ* = 220 nm, *P* = 11 mW cm^−2^) in a vacuum box. The newly fabricated device was first imaged as shown in Fig. 5a. For comparison, the response of the pristine p-MoTe_2_, n-MoTe_2_ after DUV + N_2_ treatment, and p-MoTe_2_ after DUV + O_2_ treatment under DUV light irradiation was observed in a vacuum at *V*_g_ = 0 V and *V*_ds_ = 0.5 V. When the DUV light on the pristine p-MoTe_2_ photodetector was switched ON (for 60 s), a photocurrent was generated owing to electron–hole pair generation. Then, the photocurrent quickly dropped toward its initial state when the DUV light was switched OFF (for 60 s), as shown in Fig. 5b. The photoresponse of the pristine MoTe_2_ device is shown in Fig. 5b.

Meanwhile, the self-made n-MoTe_2_ photodetector after DUV + N_2_ doping showed a faster response with higher photocurrent values, as shown in Fig. 5c. This increase in the photocurrent is likely due to the large electron density caused by DUV + N_2_ doping, which facilitates the charge carrier transport after the generation of electron–hole pairs by DUV light. Furthermore, for the self-made p-MoTe_2_ device after DUV + O_2_ doping, the photoresponse is faster but the photocurrent magnitude is reduced, as shown in Fig. 5d. The reduced current is attributed to the oxygen trap states formed by DUV + O_2_. However, the sharp rise and decay of the n-MoTe_2_ and p-MoTe_2_ photodetectors (Fig. 5c and d) are expected to occur because of the additional energy states in MoTe_2_ (n- and p-type) with N_2_ and O_2_ treatment under DUV light. When the DUV light is turned ON, an abrupt rise in the photocurrent is produced due to electron–hole pair generation, but electrons are quickly captured in trapping states of the defects and the current quickly falls down to a saturation level.

**Fig. 6:**
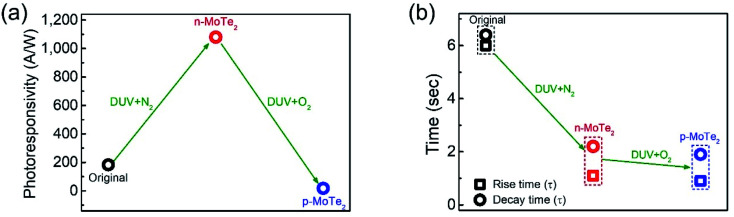
(a) Photoresponsivity of pristine p-MoTe_2_, DUV + N_2_ treated and DUV + O_2_ treated devices. (b) Response time (rise & decay) of MoTe_2_ photodetectors of each type. The rectangular shape represents the rise time constant and circle shape represents the decay time constant.

To elucidate the photoresponse in detail, we calculated the photoresponsivity of each device (Fig. 6a). The responsivities of pristine MoTe_2_, DUV + N_2_ doped MoTe_2_ (n-MoTe_2_), and DUV + O_2_ doped MoTe_2_ (p-MoTe_2_) were found to be approximately 183, 1080, and 18 A/W, respectively. This trend is expected because the DUV + O_2_ treated p-MoTe_2_ device exhibited low photocurrent generation (approximately 12 nA) compared to pristine p-MoTe_2_ (110 nA) and DUV + N_2_ doped devices (713 nA), as shown in Fig. S3.† The lower photocurrent in the DUV + O_2_ treated devices can be ascribed to the quick recombination of photogenerated charges in defect states of p-type MoTe_2_.

The response times of the pristine MoTe_2_, DUV + N_2_ doped n-MoTe_2_, and DUV + O_2_ doped p-MoTe_2_ devices were also extracted after fitting the following equations:^39,40^

3 *I*_t_ = *I*_dark_ + *A*(1 − e^−*t*/*λ*_1_^) and *I*_t_ = *I*_dark_ + *B*e^−*t*/*λ*_2_^

here, *λ*_1_ is the rise time constant, *λ*_2_ is the decay time constant, and *A* and *B* are the scaling constants used as fitting parameters. The rise/decay times of pristine p-MoTe_2_ and treated n-MoTe_2_ and p-MoTe_2_ photodetectors were estimated as 6/6.4, 1.1/2.2 and 0.9/1.9 s, respectively, as shown in Fig. 6b. The decay and rise times were reduced as the pristine p-MoTe_2_ was treated with DUV + N_2_ and DUV + O_2_, which are related to the additional defects/energy states in the mid-gap of MoTe_2_ after DUV irradiation in N_2_ and O_2_ environments.

## Conclusion

In summary, FETs were fabricated using pristine p-MoTe_2_, n-MoTe_2_ (after DUV + N_2_ treatment), and p-MoTe_2_ (after DUV + O_2_ treatment). The pristine p-MoTe_2_ gradually changed to n-MoTe_2_ after DUV + N_2_ treatment. Subsequently, the treated n-MoTe_2_ was reconfigured to p-MoTe_2_ by DUV + O_2_ treatment. We estimated that the field-effect mobility and charge carrier density of electrons/holes significantly changed under the DUV + N_2_/O_2_ treatments. In addition, we investigated each type of the MoTe_2_ photodetector at *V*_g_ = 0 V and found that the photocurrent of n-MoTe_2_ was higher than that of intrinsic p-MoTe_2_ and treated p-MoTe_2_. The DUV + O_2_ treated p-MoTe_2_ devices exhibited slightly poor performance owing to the O_2_ based electron trapping states in MoTe_2_. Interestingly, we found that the polarity of thin MoTe_2_ flakes could be easily changed from p-type to n-type and then from n-type to p-type, whereas the thicker MoTe_2_ flakes could easily transform into an n-type material after DUV + N_2_ treatment; however, their polarities were not entirely transformed from n-type to p-type after DUV + O_2_ treatment. Moreover, we found that the response time of DUV + O_2_ treated p-MoTe_2_ is faster than that of intrinsic p-MoTe_2_ and DUV + N_2_ treated n-MoTe_2_, which is attributed to the additional trapping/oxide states in the DUV + O_2_ treated p-MoTe_2_ flake. We believe that such a reversibility of carrier polarity by DUV + N_2_/O_2_ treatment can be utilized for the next generation of optoelectronic devices.

## Conflicts of interest

The authors declare no competing financial interests.

## Supplementary Material

NA-004-D1NA00881A-s001
