# Supplementary material for: Reconfigurable carrier type and photodetection of MoTe2 of various thicknesses by deep ultraviolet light illumination
Source: Nanoscale Adv. 2022 May 10;4(12):2744–51. doi: 10.1039/d1na00881a (PMC9417606; doi:10.1039/d1na00881a)
Supplement: NA-004-D1NA00881A-s001 [file NA-004-D1NA00881A-s001.pdf]

Electronic Supplementary Material (ESI) for Nanoscale Advances.
This journal is © The Royal Society of Chemistry 2022

Supporting Information

Reconfigurable carrier type and photodetection of $MoTe_2$ of various thickness by deep ultraviolet light illumination

Byung Min Ko[1#], Muhammad Farooq Khan[2#], Ghulam Dastgeer[1], Gyu Nam Han[1], Muhammad Asghar Khan[1] and Jonghwa Eom[1*]

[1]Department of Physics & Astronomy and Graphene Research Institute, Sejong University, Seoul 05006, Korea

[2]Department of Electrical Engineering, Sejong University, Seoul 05006, Korea

[#]These authors contributed equally to this work

[*]Corresponding author: eom@sejong.ac.kr

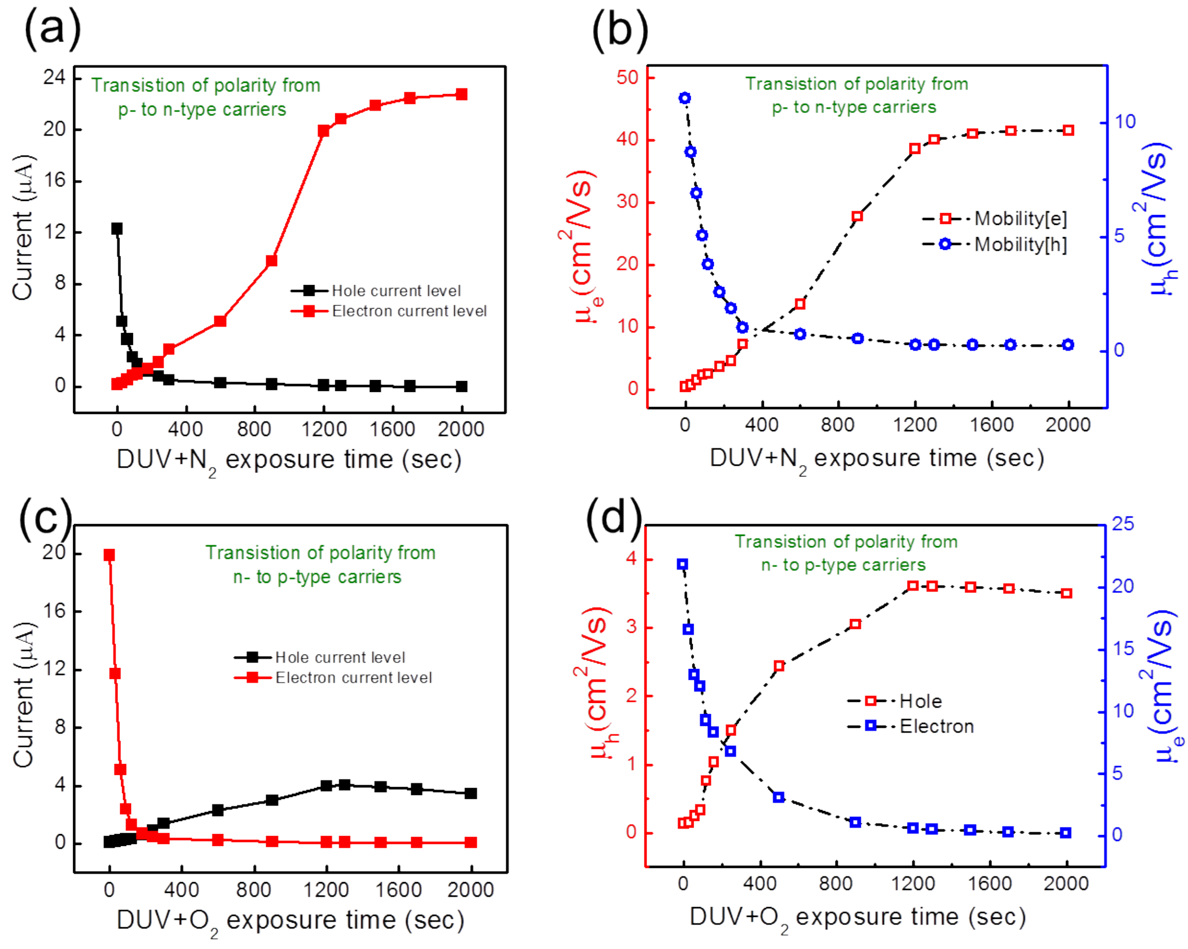

Figure S1. (a) Electron and hole current values at V_g = 60 V with different DUV+N_2 treatment times. **(b)** Electron and hole mobility at different DUV+N_2 treatment times. **(c)** Electron and hole current values at V_g = 60 V with different DUV+O_2 treatment times. **(d)** Electron and hole mobility at different DUV+O_2 treatment times.

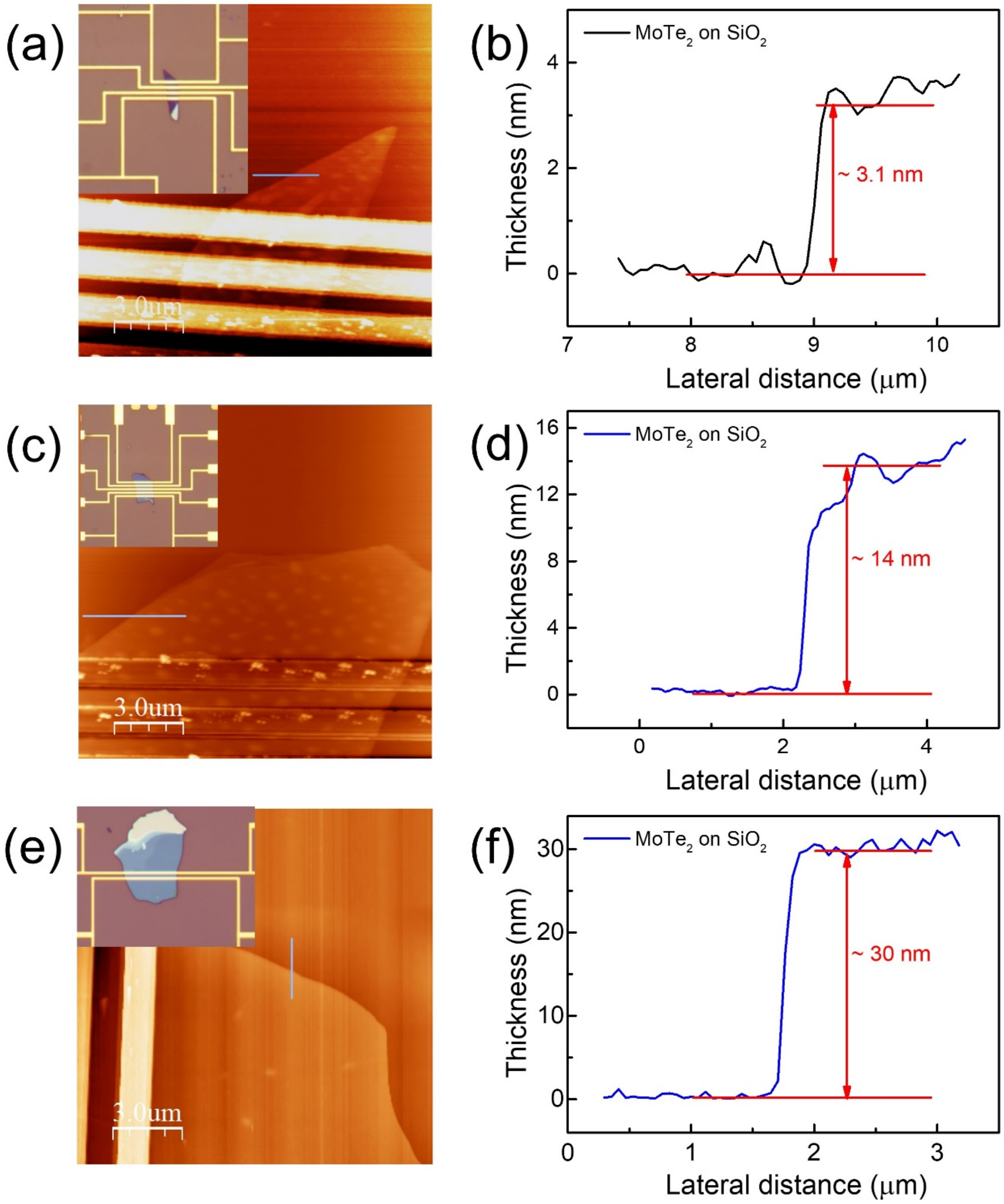

Figure S2. The AFM images and thickness profiles of different MoTe2 flakes **(a)** AFM image of 3.1-nm-thick flake. **(b)** Height profile of 3.1 nm. **(c)** AFM image of 14-nm-thick flake. **(d)** Height profile of 14 nm. **(e)** AFM image of 30-nm-thick flake. **(f)** Height profile of 30 nm.

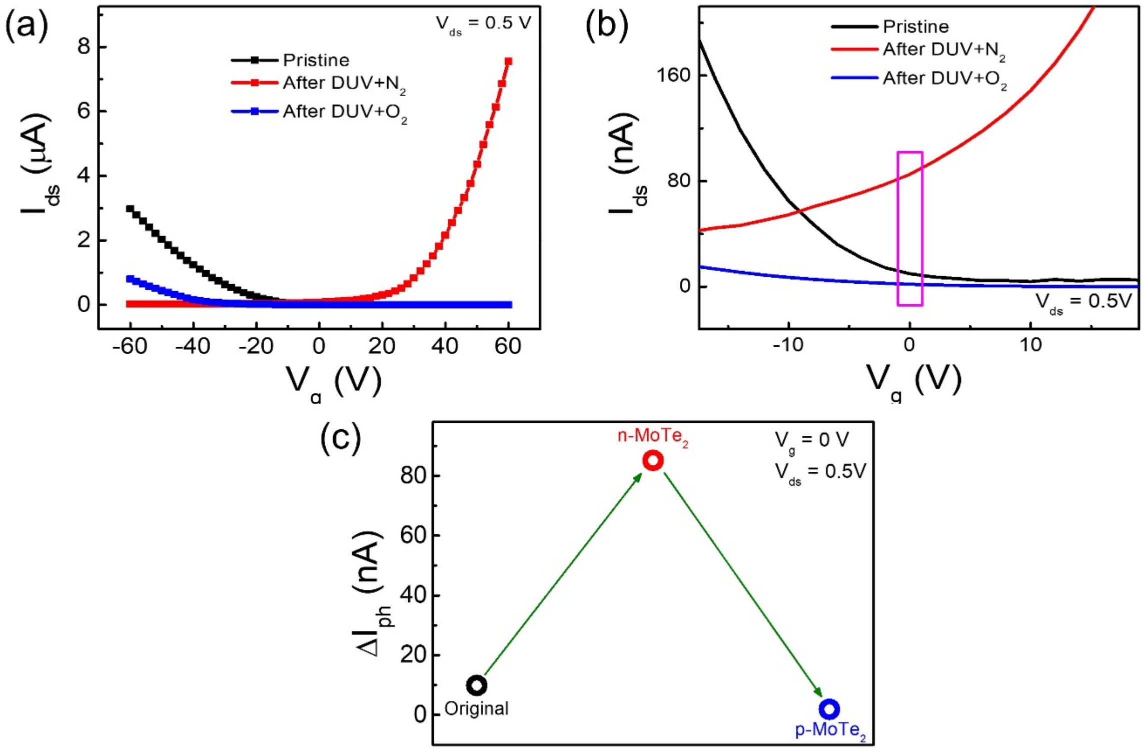

Figure S3. **(a)** Transfer curves of another pristine p-$MoTe_2$, n-$MoTe_2$ after DUV+N_2 treatment and p-$MoTe_2$ after DUV+O_2 treatment. **(b)** Zoom-in view to see the current value at V_g = 0 V. **(c)** photocurrent generation of pristine p-$MoTe_2$, n-$MoTe_2$ after DUV+N_2 treatment and p-$MoTe_2$ after DUV+O_2 treatment.
